# Sertraline safety in adolescents experiencing a depressive episode during first 3 weeks: added anxiolytic matters more than CYP2C19 polymorphisms

**DOI:** 10.3389/fphar.2026.1724521

**Published:** 2026-03-02

**Authors:** Dmitriy V. Ivashchenko, Vitaliy V. Sobur, Sergey V. Grass, Anna Y. Basova, Rimma V. Kondratieva, Eugenia N. Shagovenko, Yulia V. Chernetsova, Svetlana N. Tuchkova, Ivan N. Korsakov, Sergey I. Markov, Karin B. Mirzaev, Yuriy S. Shevchenko, Dmitry Sychev

**Affiliations:** 1 Federal State Budgetary Research Institution Russian Research Center of Surgery Named After Academician B.V. Petrovsky, Moscow, Russia; 2 Russian Medical Academy of Continuous Professional Education, Moscow, Russia; 3 Scientific-Practical Children’s and Adolescents Mental Health Center n.a. G.E. Sukhareva, Moscow, Russia

**Keywords:** CYP2C19, depression, gene, genetics, genome, genotype, safety, sertraline

## Abstract

**Objectives:**

To evaluate the associations of clinical and pharmacogenetic factors (CYP2C19*2, *17 polymorphisms) with the safety parameters of sertraline in adolescents with a depressive episode and suicidal intentions for 3 weeks in a psychiatric hospital.

**Methods:**

The study included 112 adolescents, who were hospitalized due to a depressive episode and suicidal intentions. All patients received sertraline for 21 days. The safety of pharmacotherapy was assessed on the 7th and 21st day using the Antidepressant ADRs checklist. Each patient underwent genetic testing for CYP2C19*2, *3, *17. The safety of sertraline was analyzed depending on the carrier status of *CYP2C19* polymorphisms, the type of CYP2C19 metabolism, as well as depending on additional pharmacotherapy.

**Results:**

It was found that patients with the CYP2C19*2 genotype took a slightly lower dose of sertraline compared to those with the wild-type genotype on 21st day (100 [87.5; 100] mg/day vs. 100 [100; 100], p = 0.048). Patients carrying the CYP2C19*17 allele, on the other hand, were more likely to experience vegetative and somatic ADRs on day 7 of treatment (2 vs. 1, p = 0.042). The use of anxiolytics (alimemazine and hydroxyzine) was significantly associated with an increased number of vegetative/somatic, and mental ADRs on day 7, as well as an increased total number of ADRs by day 21. Linear regression analysis confirmed that anxiolytics were the most significant predictors of ADR development on day 21 (Estimate = 0.86, 95% CI 0.32-1.41, p = 0.002).

**Conclusion:**

Polypharmacy (particularly, adding a non-benzodiazepine anxiolytic) was a more significant risk factor for ADRs when taking sertraline, compared to the carrier status of CYP2C19 gene polymorphisms.

## 1 Introduction

Selective serotonin reuptake inhibitors (SSRIs) can cause adverse reactions in 5%–32% of children (Rynn et al., 2015). Only 35% of patients respond to the first prescribed antidepressant, and 40% develop drug-resistant depression (Souery et al., 2011). The most commonly used drugs are citalopram, escitalopram, sertraline, and fluoxetine (Lukmanji et al., 2020). In Russia, the use of sertraline is officially allowed from the age of 6, fluvoxamine from the age of 8, so these antidepressants are most frequently prescribed to children.

Children are not only more likely to have adverse drug reactions (ADRs) to SSRIs compared to adults (31.7% vs. 23.1%, p < 0.05) (Rynn et al., 2015), they have their own special range of ADRs to antidepressants (Cipriani et al., 2016). Increased suicidal thoughts (1%–4%) (Lagerberg et al., 2022), activation (до 45%) (Strawn et al., 2023), nausea (до 22%) (Strawn et al., 2023) are the most common adverse reactions in the first weeks of taking SSRIs. In adolescents, the risk of suicidal thoughts and behavior increases more than twofold (Sharma et al., 2016).

The existing problems of the safe administration of antidepressants in children and adolescents require active study of the personalized selection of pharmacotherapy. Pharmacogenetics is currently the most accessible and studied personalization technology (Bousman et al., 2023).

Currently, there are comprehensive solutions available for selecting an antidepressants for patients based on pharmacogenetic testing. Several meta-analyses have been conducted to compare the efficacy and safety of pharmacological treatment for depressive episodes, taking into account the use of personalized algorithms (Brown et al., 2022; Arnone et al., 2023; Bunka et al., 2023; Wang et al., 2023; Milosavljević et al., 2024). However, despite the positive findings (the use of personalized algorithms has increased the efficacy of antidepressant treatment), those studies did not provide sufficient evidence to fully implement these algorithms in clinical practice (Milosavljević et al., 2024). This is due to the fact that different algorithms are used all over the world, and the effect size of their use is relatively small (Milosavljević et al., 2024).

In 2023, the recommendations of the Clinical Pharmacogenetic Implementation Consortium (CPIC) for the personalized selection of sertraline based on pharmacogenetic testing of *CYP2C19* and *CYP2B6* in adults were published (Bousman et al., 2023). To date, there is a lot of convincing evidence that the genetically determined type of CYP2C19 metabolism affects the efficacy and safety of sertraline in adults (Milosavljević et al., 2024). A recent large-scale study by Eijsbouts C. et al. (2024), which involved more than 114,000 participants, found that individuals who are “poor” CYP2C19 metabolizers are significantly more likely to experience tremors and sexual dysfunction while taking sertraline (Eijsbouts et al., 2024).

However, in adolescents and young adults, the findings from SSRI pharmacogenetic studies are less promising. In a study conducted by Strawn et al. (2020) on adolescents (n = 26) with generalized anxiety disorder who were taking escitalopram, genotyping of the *CYP2C19* gene and steady-state concentrations were measured. The results confirmed the impact of the *CYP2C19* genotype on the pharmacokinetics of escitalopram in this age group (Strawn et al., 2020). No effect on efficacy and safety has been identified, which could have been due to small sample size (Strawn et al., 2020). A study by Poweleit et al. (2019) found no significant associations between *CYP2C19* polymorphisms and sertraline intake outcomes in adolescents with anxiety and depressive disorders (Poweleit et al., 2019). Finally, a large-scale study on the pharmacogenetic basis of the safety of sertraline and escitalopram in adolescents has led to paradoxical findings (Rossow et al., 2020). Adolescents with “normal” CYP2C19 metabolism were more likely to experience adverse reactions compared with “poor” metabolizers (Rossow et al., 2020). This contradicts the findings that were previously published for adult patients (Bousman et al., 2023; Milosavljević et al., 2024).

A prospective comparative study on the algorithm for personalized therapy and the empirical selection of an antidepressant for adolescents with a depressive episode has not revealed the benefits of pharmacogenetic testing (Vande Voort et al., 2022). Thus, the development of new algorithms for the personalization of SSRIs based on separate studies is required for adolescents.

A related issue is that pharmacogenetic testing cannot be generalized to other ethnic populations without data on the predictive value of biomarkers for that specific population (Roberts et al., 2023). In particular, the prevalence of CYP2C19*2 and *3 polymorphisms is significantly higher among Asians than among Caucasians (Biswas et al., 2023; Nakhonsri et al., 2024). In contrast, CYP2C19*17 is more common among Caucasians (Mirzaev et al., 2019). Many ethnic groups live in Russia, and significant differences in the frequency of *CYP2C19* polymorphisms have also been found among them (Mirzaev et al., 2019; Mirzaev et al., 2020; Abdullaev et al., 2020). It is, therefore, essential to conduct research on Russian patients. Local studies in adult patients with depressive episodes comorbid with alcoholism have found significant associations between the efficacy and safety of SSRIs and the cytochrome P450 (CYP) enzymes CYP2D6 and CYP2C19 (Zastrozhin et al., 2021a; Zastrozhin et al., 2021b).

The aim of this study was to evaluate the associations of carrier status of CYP2C19*2, *17 with the efficacy and safety parameters of sertraline in adolescents with a depressive episode and suicidal intentions for 3 weeks in a psychiatric hospital.

## 2 Materials and methods

The study was conducted in accordance with the Declaration of Helsinki, and approved by the local ethics committee of the “Scientific-Practical Children’s and Adolescents Mental Health Center n. a. G.E. Sukhareva (Minute No. 2/23 dated 05/17/2023).

Study design: observational naturalistic. The study involved patients admitted to the “Scientific-Practical Children’s and Adolescents Mental Health Center n. a. G.E. Sukhareva” from 20 May 2023 to 31 August 2024.

The medical records of children admitted to inpatient treatment were examined for compliance with the inclusion and exclusion criteria. A total of 112 adolescents were followed up for 3 weeks.

Inclusion criteria:

• Age from 12 to 17 years inclusive.
• Depressive syndrome as the main reason for treatment.
• Suicidal tendencies in the patient (thoughts, preparations, or attempts).
• Prescribing of sertraline.
• Signed voluntary informed parental consent for the patient’s participation in the study.

Exclusion criteria:

• Diagnosis of bipolar affective disorder (F31. X by ICD-10).
• Diagnosis of schizophrenic spectrum disorders (F2X by ICD-10).
• Non-compliance with the inclusion criteria.
• Refusal to participate in the study.

The inclusion was performed on the first day of the patient’s admission to a psychiatric hospital. In each case, the patient’s legal representative signed an informed voluntary consent to participate in the study. Each patient received an age-appropriate explanation of the study aim, their involvement in it, and the procedures that were to experience while participating in the study. Each adolescent also signed an informed voluntary consent to participate in the study. The personal data that could lead to the identification of the patient was not entered into electronic databases.

The patients were included in the stufy on the first day of their admission to a psychiatric hospital. In each case, the patient’s legal representative signed an informed voluntary consent form to participate in the study. The personal data that could lead to the identification of the patient was entered into electronic databases.

### 2.1 Clinical and demographic characteristics of patients

The individual registration chart for each patient included: gender, age, height, weight, body mass index (BMI), main diagnosis, total number of hospitalizations, age of onset of symptoms of the mental disorder, history of suicide attempt, the fact of non-suicidal self-harm, age of occurrence of non-suicidal self-harm (if any), age of the first suicide attempt (if available), the total number of suicidal attempts, the current presence of suicidal thoughts, the fact of taking an antidepressant immediately before hospitalization.

### 2.2 Pharmacotherapy

All patients were taking sertraline as their primary therapy. Some patients were additionally prescribed antipsychotics, mood stabilizers, anticholinergic drugs to remedy extrapyramidal symptoms, and anxiolytics. Such cases were considered as polypharmacy and were necessarily taken into account in the analysis.

The rater could not influence the prescription of psychopharmacotherapy by the attending physician. All psychotropic drugs received by the patient were entered into an individual registration chart. The daily doses of medications taken at the time of inclusion, on the 7th and 21st days of follow-up were taken into account.

### 2.3 Assessment of pharmacotherapy safety

In a hospital setting, the effectiveness and safety of the therapy received was evaluated on day 7 and 21.

The “Antidepressant adverse drug reactions checklist” [adopted from Rossow et al. (2020)] was used to assess the safety of pharmacotherapy over time.

Based on this checklist, we identified two categories of symptoms: (1) psychiatric and (2) somatic and vegetative. In our analysis, we considered both the overall sum of symptoms within these two categories and the overall total number of symptoms. Additionally, we performed a comparative analysis of the occurrence frequency of individual symptoms based on the responses to this questionnaire.

### 2.4 Genetic analysis

On the day of inclusion in the study, 5 mL of blood were collected from each patient in disposable sterile vacuum tubes with EDTA, for subsequent genotyping. The biomaterial was taken simultaneously with routine tests and did not require additional venipunctures. The blood was frozen at −20° C, transported to the laboratory and subsequently stored at −70° C.

The laboratory part of the study was conducted on the basis of the Research Institute of Molecular and Personalized Medicine of the Russian Medical Academy of Continuous Professional Education (Moscow). DNA isolation and genotyping of the samples took place as they were received between 1 June 2023 and 31 August 2024.

DNA was isolated from venous blood using the column method using the QIAamp DNA Blood Mini Kit (Qiagen, Germany). The concentrations and quality assessment of the obtained DNA preparations were carried out using a Qubit 4 fluorimeter (Thermo Fisher Scientific, United States) and a Nanodrop ND-1000 spectrophotometer (Thermo Fisher Scientific, United States).

Genetic polymorphisms CYP2C19*2 (rs4244285 G681A), CYP2C19*3 (rs4986893 G636A), CYP2C19*17 (rs12248560 C-806T) were determined by real-time polymerase chain reaction (PCR) using commercial reagent kits, equipment: CFX96 TouchTM Real-Time PCR Detection System (Bio-Rad, United States).

### 2.5 Statistical analysis

Statistical analysis was carried out using the SPSS Statistics 26.0. We did not perform a preliminary sample size calculation. The preliminary sample size was based on the number of patients in previous studies on pharmacogenetic risk factors for adverse reactions to antidepressants (Amitai et al., 2016; Poweleit et al., 2019; Strawn et al., 2019; Rossow et al., 2020; Honeycutt et al., 2024). Due to the abnormal distribution of data, nonparametric criteria were used to compare quantitative variables between groups. The results of calculations of quantitative variables were presented as median and quartiles - Me [Q1; Q3].

All patients were divided into subgroups according to the genotypes of the polymorphisms: carriers of the polymorphic allele (heterozygotes + homozygotes) and homozygotes for the “wild” allele. For example, carriers of the CYP2C19*2 were divided into two subgroups: GG and GA + AA. Subsequently, the genetically determined subgroups were compared to find associations with the clinical parameters of the patients.

According to the results of pharmacogenetic testing, the type of CYP2C19 metabolism was determined for each patient according to the Dutch Pharmacogenetics Working Group algorithm: “ultrarapid”, “normal”, “intermediate”, “poor” (Brouwer et al., 2022).

The Mann-Whitney criterion was used to compare the selected subgroups at the same time point according to quantitative variables. The frequencies of categorical variables were compared with each other using Pearson’s Chi-square, and Fisher’s exact criterion was used for 2x2 comparisons. The calculation of the correspondence of the genotype distribution to the Hardy-Weinberg law was performed using an online calculator (Hardy-Weinberg Calc, 2025).

Linear regression was performed using the least squares method to identify the most significant risk factors for ADRs when taking sertraline.

When analyzing the data, the influence of demographic and clinical characteristics of patients on the studied outcomes, including the effect of polypharmacy, has always been taken into account. This was done in order to establish the significance of associations of *CYP2C19* gene polymorphisms with the parameters of sertraline safety.

## 3 Results

### 3.1 Sample description

The demographic and clinical characteristics of the participants in the study are presented in Table 1.

**TABLE 1:** Clinical and demographic characteristics of the sample.

Variables	All participants (n = 112)
Age, years (Me [Q1; Q3])	15 [14; 16]
Height, m (Me [Q1; Q3])	1,64 [1,58; 1,68]
Weight, kg (Me [Q1; Q3])	54,15 [47; 62,85]
BMI (Me [Q1; Q3])	19,97 [18,3; 22,3]
Female (n, %)	101, 90,2%
Total number of hospitalizations (including the current one) (Me [Q1; Q3])	1 [1; 1]
Age of onset of symptoms of mental disorder, years (Me [Q1; Q3])	13 [12; 14]
Suicidal thoughts (n, %)	105, 93,8%
A history of NSSI (n, %)	104, 92,9%
Age of occurrence of NSSI, years (Me [Q1; Q3])	14 [12,25; 14]
History of suicide attempt (n, %)	48, 42,9%
Total number of suicide attempts (Me [Q1; Q3])*	0 [0; 1]
Age at the start of taking antidepressants, years (Me [Q1; Q3])	15 [14; 16]
Duration of mental disorder before inclusion in the study, months (Me [Q1; Q3])	18,5 [9; 27]

BMI, body mass index, * - n = 48.

The average age of the participants was 15 years [14; 16]. Of these, there were 101 females (90.2%). Among all participants, 93.8% reported having experienced suicidal ideation, 92.9% had engaged in self-harm behavior, and 42.9% had a history of attempted suicide.

The genotype frequencies for the polymorphisms CYP2C19*2 and CYP2C19*17 in the study population were as follows: CYP2C19*2 (GG = 85, GA = 25, AA = 2) and CYP2C19*17 (CC = 69, CT = 37, TT = 6). No significant deviations from Hardy-Weinberg equilibrium were observed.

Of the 112 subjects, 78 displayed “normal” CYP2C19 metabolic activity, 26 displayed “intermediate” metabolic activity, and six displayed “ultrarapid” metabolic activity. Due to the small number of subjects with “ultrarapid” (n = 6) or “poor” (n = 2) metabolic phenotypes, these patients were excluded from further analysis.

Table 2 presents the results of a comparative analysis of the clinical and demographic characteristics of patients, based on their carriage of *CYP2C19* gene polymorphisms, as well as their isoenzyme metabolic phenotype (between “normal” and “intermediate”).

**TABLE 2:** Demographic and clinical characteristics of the sample depending on the carriage of polymorphic variants of CYP2C19*2, CYP2C19*17, type of CYP2C19 metabolism.

Variables	CYP2C19*2		p	CYP2C19*17		p	CYP2C19 metabolism		p
	GG (n = 85)	GA + AA (n = 27)		CC (n = 69)	CT + TT (n = 43)		Normal (n = 78)	Intermediate (n = 26)	
Age, years (Me [Q1; Q3])	15 [14; 16]	14 [13,2; 15]	0.112	15 [14; 16]	14 [14; 15]	0.348	15 [14; 16]	14 [14; 15]	0.157
Height, m (Me [Q1; Q3])	1.64 [1.59; 1.69]	1.62 [1.58; 1.68]	0,5	1.64 [1.58; 1.68]	1.64 [1.59; 1.69]	0.728	1.64 [1.59; 1.68]	1.63 [1.58; 1.68]	0.646
Weight, kg (Me [Q1; Q3])	54.25 [49; 63,7]	49,1 [43,62; 61,13]	0.241	54.4 [48.42; 63.5]	53,5 [46,3; 61]	0.225	54.35 [49.53; 64.0]	51.0 [44.0; 63.0]	0.390
BMI (Me [Q1; Q3])	20.18 [18.61; 23,25]	19.05 [17.44; 21.24]	0.118	19.88 [18,44; 22,42]	20 [17,47; 22,21]	0.204	20.62 [18,76; 23.39]	19.15 [17,48; 21,5]	0.164
Total number of hospitalizations (including the current one) (Me [Q1; Q3])	1 [1; 1]	1 [1; 1]	0.419	1 [1; 1]	1 [1; 1]	0.398	1 [1; 1]	1 [1; 1]	0.454
Age of onset of symptoms of mental disorder, years (Me [Q1; Q3])	13 [12; 14]	13 [12; 14]	0.582	13 [12; 14]	13 [12; 14]	0.567	13 [12; 14]	13 [12; 14]	0.570
Age of occurrence of NSSI, years (Me [Q1; Q3])	14 [13; 14]	13 [12; 14.75]	0.691	14 [12,5; 15]	13 [12; 14]	0.316	14 [13; 14]	13 [12; 15]	0.556
Total number of suicide attempts (Me [Q1; Q3])^a^	0 [0; 1]	0 [0; 1]	0.96	0 [0; 1]	0 [0; 1]	0.814	0 [0; 1]	0 [0; 1]	0.878
Age at the start of taking antidepressants, years (Me [Q1; Q3])	15 [14; 16]	14,5 [13,25; 15]	0.422	15 [14; 16]	14 [14; 15]	0.132	15 [14; 16]	15 [14; 15]	0.489
Duration of mental disorder before inclusion in the study, months (Me [Q1; Q3])	19,5 [10,25; 27]	14 [5.25; 31,5]	0.5	18 [8,5; 28,5]	20 [10; 27]	0.864	18 [10; 27]	16 [5; 32]	0.724
Male, n (%)	6 (7,1%)	5 (18,5%)	0.081	10 (14,5%)	1 (2,3%)	0.035	6 (7,7%)	5 (19,2%)	0.137
Female, n (%)	79 (92,9%)	22 (81,5%)		59 (85,5%)	42 (97,7%)		72 (92,3%)	21 (80,8%)	
Suicidal thoughts, n (%)	78 (91,8%)	27 (100%)	0.124	64 (92,8%)	41 (95,3%)	0.581	72 (92,3%)	26 (100%)	0.333
A history of NSSI, n (%)	79 (92,9%)	22 (92,6%)	0.821	66 (95,6%)	39 (90,7%)	0.428	74 (94,9%)	24 (92.3%)	0.756
History of suicide attempt, n (%)	37 (43,5%)	11 (40,7%)	0.799	30 (43,5%)	18 (41,9%)	0.866	34 (43,6%)	11 (42,3%)	0.909

BMI, body mass index.

^a^ n = 48.

The study found that in our sample, the majority of carriers of the CYP2C19*17 polymorphism were female, with only one male carrier of the T allele identified (p = 0.035). All other parameters were comparable between the samples.

### 3.2 Analysis of psychopharmacotherapy depending on the carriage of *CYP2C1*9 polymorphisms

Table 3 presents the results of a comparative analysis of psychopharmacotherapy in patients.

**TABLE 3:** The results of the analysis of psychopharmacotherapy depending on the carriage of polymorphic variants of CYP2C19*2, CYP2C19*17, and type of CYP2C19 metabolism.

Variables	Total sample (n = 112)	CYP2C19*2		p	CYP2C19*17		p	CYP2C19 metabolism		p
		GG (n = 85)	GA + AA (n = 27)		CC (n = 69)	CT + TT (n = 43)		Normal (n = 78)	Intermediate (n = 26)	
Sertraline starting dose, mg/day (Me [Q1; Q3])	25 [25; 25]	25 [25; 25]	25 [25; 37,5]	0.807	25 [25; 25]	25 [25; 25]	0.922	25 [25; 25]	25 [25; 25]	0.826
The dose of sertraline on day 7, mg/day (Me [Q1; Q3])	75 [75; 75]	75 [56,25; 75]	75 [75; 75]	0.545	75 [75; 75]	75 [75; 75]	0.672	75 [75; 75]	75 [75; 75]	0.934
The dose of sertraline on day 21, mg/day (Me [Q1; Q3])	100 [100; 100]	100 [100; 100]	100 [87,5; 100]	0.048	100 [100; 100]	100 [100; 100]	0.831	100 [100; 100]	100 [100; 100]	0.245
Antipsychotic, n (%)	64 (57,1%)	49 (57,6%)	15 (55,6%)	0.848	39 (56,5%)	25 (58,1%)	0.866	47 (60.26%)	13 (50.00%)	0.492
Mood stabilizer, n (%)	15 (13,4%%)	10 (11,8%)	5 (18,5%)	0.369	11 (15,9%)	4 (9,3%)	0.316	10 (12.82%)	5 (19.23%)	0.629
Anxiolytic, n (%)	50 (44,6%)	39 (45,9%)	11 (40,7%)	0.64	31 (44,9%)	19 (44,2%)	0.939	35 (44.87%)	11 (42.3%)	0.985
Anticholinergic drug, n (%)	18 (16,1%)	13 (15,3%)	5 (18,5%)	0.691	12 (17,4%)	6 (14,0%)	0.63	12 (15.38%)	3 (11,53%)	0.872

The majority of patients (57.1%) received an antipsychotic in addition to their treatment, most commonly quetiapine (n = 19), paliperidone (n = 14), and risperidone (n = 12). Aripiprazole was prescribed in five cases. The remaining antipsychotic medications were prescribed in fewer than three cases each: lurazidone, olanzapine, periciazine, perphenazine, thioridazine, and chlorpromazine. Carbamazepine was given to nine patients, lamotrigine to five, and oxcarbazepine in one case. Anxiolytics were given to 50 patients (44.6%) and were all histamine receptor antagonists, namely, alimemazine in 30 cases and hydroxyzine in 20. In addition, biperiden, an anticholinergic medication, was given to 18 patients to correct extrapyramidal symptoms associated with the use of antipsychotics.

A comparative analysis of carriers of different *CYP2C19* polymorphisms revealed that on day 21 carriers of the CYP2C19*2 took a slightly lower dose of sertraline (100 [87.5; 100] vs. 100 [100; 100] mg/day; p = 0.048).

### 3.3 Associations of *CYP2C19* polymorphisms with safety parameters of sertraline in adolescents

**FIGURE 1:**
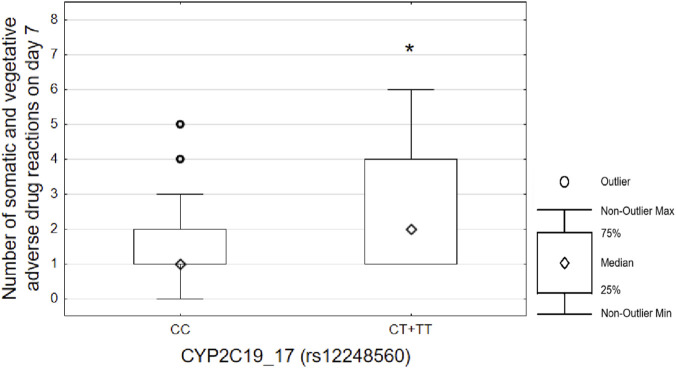
Number of somatic and vegetative adverse drug reactions in carriers of CYP2C19*17 polymorphism. Notes: carriers of CYP2C19*17 (CT + TT genotypes) had more somatic and vegetative adverse drug reactions on day seven (2 [1; 3] vs. 1 [1; 2]; p = 0.042).

Carriers of CYP2C19*17 (CT + TT genotypes) were more likely to complain of somatic/vegetative adverse reactions on day 7 (2 [1; 3] vs. 1 [1; 2]; p = 0.042). Results are shown on Figure 1. No other significant associations were identified.

### 3.4 Analysis of associations of the prescription of additional pharmacotherapy with the safety parameters of sertraline

**FIGURE 2:**
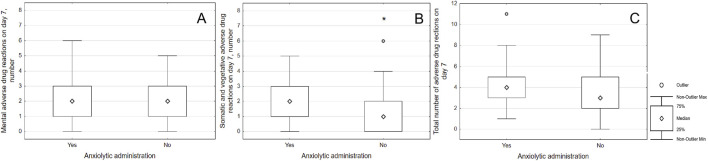
Associations of the number of adverse drug reactions with administration of anxiolytic on day seven. Notes: (*) means significant differences, patients who were prescribed an anxiolytic were more likely to have vegetative and somatic ADRs **(B)** on the 7th day of follow-up (2 [1; 3] vs. 1 [0; 2]; p = 0.033). There were no significant difference in mental ADRs **(A)** or total number of ADRs **(C)**.

**FIGURE 3:**
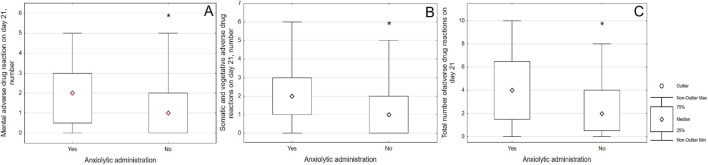
Associations of the number of adverse drug reactions with administration of anxiolytic on day twenty one. Notes: (*) means significant differences; patients who were prescribed an anxiolytic were more likely to have mental ADRs **(A)** on day 21 (2 [1; 3] vs. 1 [0; 2]; p = 0.015), vegetative and somatic ADRs **(B)** (2 [1; 3] vs. 1 [0; 2]; p = 0.003), as well as a large total number of ADRs **(C)** (4 [1.5; 6.5] vs. 2 [0.5; 4]; p = 0.003).

The frequency of ADRs was assessed depending on the prescription of additional psychopharmacotherapy. When prescribing a tranquilizer, only vegetative and somatic ADRs were detected on day 7 (2 [1; 3] vs. 1 [0; 2]; p = 0.033); but on the 21st day there was an increase in the total number of ADRs (4 [1.5; 6.5] vs. 2 [0.5; 4]; p = 0.003), the numbers of somatic and vegetative ADRs (2 [1; 3] vs. 1 [0; 2]; p = 0.003) and mental ADRs numbers (2 [1; 3] vs. 1 [0; 2]; p = 0.015). Results were illustrated on Figures 2, 3.

The administration of other drugs did not significantly associated with incidence of ADRs in patients.

The administration of an additional antipsychotic was associated with complaints of flatulence (10.9% vs. 0%; p = 0.019) on day 7 of follow-up. On day 21, taking an antipsychotic was associated with complaints of drowsiness (41.3% vs. 20.9%; p = 0.029).

Adolescents who took a anxiolytic were significantly more likely to express homicidal thoughts (8.0% vs. 0%; p = 0.037) and complaints of increased appetite (18.0% vs. 1.6%; p = 0.005) on day 7. On day 21, taking an anxiolytic was associated with increased complaints of unusual dreams (37.8% vs. 19.8%; p = 0.039), dizziness (26.7% vs. 11.5%; p = 0.044), headache (17.8% vs. 4.9%; p = 0.032), constipation (11.1% vs. 1.6%; p = 0.037) and tremor (31.1% vs. 14.8%; p = 0.043).

The prescription of an antiholinergic drug was associated with complaints of decreased libido on the 21st day of follow-up (16.7% vs. 3.4%; p = 0.027).

### 3.5 Linear regression analysis

A linear regression analysis was performed to evaluate significant predictors of ADRs development when taking sertraline. The dependent variables were: the number of adverse reactions according to the patient’s survey on days 7 and 21. The following covariates were selected: age, BMI, CYP2C19*2, CYP2C19*17, the fact of an antipsychotic prescription, the fact of a mood stabilizer prescription, the fact of prescribing a tranquilizer, the fact of prescribing an antichonergic drug.

Statistically significant regression analysis results are presented in Table 4.

**TABLE 4:** Linear regression analysis: significant predictors of the number of adverse reactions on days 7 and 21 of follow-up.

Outcome	Covariates	Estimate (regression coefficient)	Lower 95%	Upper 95%	p-value
Vegetative and somatic ADRs number on day 7	Anxyolitic	0.376	0.108	0.644	0,0064
​	CYP2C19*17 CT + TT	0.262	0.001	0.523	0.048
Mental ADRs number on day 21	Anxyolitic	0.476	0.158	0.795	0,0037
Vegetative and somatic ADRs number on day 21	Anxyolitic	0.384	0.049	0.719	0.024
Total ADRs number on day 21	Anxyolitic	0,86	0.319	1,403	0,0022

ADRs–adverse drug reactions.

Linear regression analysis confirmed the results obtained from previous comparisons. Carriage of the *CYP2C19* polymorphisms was weakly associated with somatic/vegetative ADRs on day 7 of sertraline administration. Anxiolytics use was associated with ADRs on days 7 and 21 of follow-up. The strongest association with anxiolytic administration was noted for the total number of ADRs on day 21.

## 4 Discussion

We conducted a study to investigate the association between the carrier status of CYP2C19*2 and CYP2C19*17 polymorphisms and the safety parameters of sertraline treatment initiation in adolescents with depressive episodes. All adolescents included in this study exhibited suicidal intentions. In our study, we took into account the demographic and clinical factors, which allowed us to objectively evaluate the contribution of pharmacogenetic biomarkers.

A comparative analysis of clinical and demographic characteristics revealed a few significant differences between individuals with different polymorphisms of the *CYP2C19* gene. First of all, differences in the frequency of CYP2C19*17 between boys and girls were identified. Our study mainly included girls, and the almost complete absence of boys who are carriers of CYP2C19*17 significantly limits the possibility of extrapolating the results to other samples. Thus, when we talk about the associations of CYP2C19*17 with clinical outcomes in our study, we can only talk about girls. Another finding was that there was a lower sertraline dose on day 21 for individuals who were carriers of the CYP2C19*2 polymorphism. This polymorphism is associated with “poor” metabolism of the enzyme, and it can be hypothesized that the poorer tolerance of sertraline leads to a lower daily dose. However, this hypothesis was not supported by the results of the patient survey, as the frequency of ADRs on day 21 did not differ significantly between those who carried the CYP2C19*2 (GA + AA genotypes) and those who had the GG wild-type genotype.

A distinctive feature of our study is the observation period of up to 3 weeks. We evaluated only the period of initiation of sertraline therapy in adolescents with depressive episodes, which is poorly represented in the literature. Most studies evaluate a period of 4 weeks or more (Poweleit et al., 2019; Strawn et al., 2020; Strawn et al., 2023). Meanwhile, adverse reactions often occur in the first weeks of SSRI treatment and may be forgotten by the patient when questioned at 4–8 weeks.

The only association between ADRs and pharmacogenetic factors was observed for CYP2C19*17. During the 7-day follow-up period, individuals carrying CYP2C19*17 were more likely to experience vegetative and somatic ADRs. It should be noted that sertraline dosage did not differ among individuals with different CYP2C19*17 genotypes, and there were no differences in the prescription of additional pharmacotherapy.

Since the carrier status of the CYP2C19*17 allele is not associated with a reduced rate of sertraline metabolism, this finding appears to be paradoxical. Similar observations have been described by Rossow et al. (2020), who noted that “normal” CYP2C19 metabolizers experienced more ADRs when taking sertraline compared to patients with a “poor” metabolic profile (Rossow et al., 2020).

But in the study by Rossow et al. (2020), there was a long period of retrospective evaluation. In our study, we found a significant association between CYP2C19*17 polymorphism and ADRs only on day 7, and no association on day 21.

It is worth noting the study by Poweleit et al. (2019), which examined the association between the carrier status of *CYP2C19* gene polymorphisms and sertraline dosing, and the incidence of ADRs in adolescents. The researchers found that there was no significant association between CYP2C19 genetic variants and sertraline’s safety profile over a 90-day period (Poweleit et al., 2019). However, the sertraline dosage was significantly lower for “poor” metabolizers, which is an indirect indicator of the poorer tolerance of the medication. In our study, similar results were obtained for CYP2C19*2 carriers. Meanwhile, patients did not differ in the number of complaints regarding ADRs depending on their CYP2C19*2 carrier status. However, Poweleit et al.'s (2019) work did not include an assessment of early ADRs, as the authors only assessed patient conditions on days 30, 60, and 90 (Poweleit et al., 2019). This prevents us from fully comparing the results of the study conducted by Poweleit et al. (2019) to our own study.

The reason for the increased frequency of ADRs among carriers of CYP2C19*17 remains unclear. Interestingly, the statistical significance of the association between sertraline metabolic rate and safety disappeared by day 21. Therefore, it is important to consider the effect of sertraline’s metabolism on safety in the initial days of treatment. By day 21, patients may have adapted to the medication. Due to the lack of significant differences in dosage based on CYP2C19*17 polymorphism, it is not possible to discuss the effect of various sertraline dosages on tolerability. It is proposed that the increased complaints of ADRs during the first week of sertraline treatment among CYP2C19*17 carriers may be associated with withdrawal symptoms. It is hypothesized that accelerated sertraline metabolism may cause withdrawal symptoms during the interval between doses (Strawn et al., 2023). The symptoms of antidepressant discontinuation syndrome are not specific and may not be recognized as such by patients. However, after 21 days, a steady-state concentration of sertraline has been established, and the withdrawal syndrome has leveled off. Patients report discomfort less frequently. Nevertheless, it is still unclear why carriers of CYP2C19*2 and “intermediate” metabolizers in this study did not experience an increase in ADRs. Therefore, our findings differ from previous studies conducted on adult patients (Milosavljević et al., 2021; Eijsbouts et al., 2024).

Adolescents who received additional medication in addition to their primary treatment, experienced a significantly higher number of ADRs in the vegetative and somatic systems in the initial stages of treatment. Furthermore, the number of complaints of all kinds increased after 21 days, which may be attributed to the cumulative effects of the medications or the potential impact of the additional pharmacotherapy on the metabolism of sertraline (phenoconversion).

The administration of anxiolytics (alimemazine and hydroxyzine, in our study) was accompanied by an increased incidence of ADRs, especially on day 21. Previous studies on hydroxyzine in children have identified sedation as the most common ADR (Miligkos et al., 2021). In general, hydroxyzine is considered a safe drug in children (Ghanizadeh and Zare, 2013; Miligkos et al., 2021), although there are concerns about its effect on psychomotor development (Gober et al., 2022). Alimemazine has been studied less thoroughly, but there are descriptions of extrapyramidal disorders induced by it (Van Maldegem et al., 2002; Gonzalez et al., 2017). We have not identified any studies on the concurrent use of sertraline and hydroxyzine or alimemazine, which our study could be compared to. The administration of anxiolytic medications was associated with an overall increase in ADRs, with the majority of effects occurring on day 21. The following adverse events were significantly associated with anxiolytic use: unusual dreams, dizziness, headache, constipation, and tremor. Given that antihistamine medication studies have generally not reported ADRs, further research is needed to better understand the potential interactions between these medications (Ghanizadeh and Zare, 2013; Miligkos et al., 2021). We can conclude that the use of antihistamine anxiolytics in combination with sertraline in adolescents increases the risk of ADRs. At the same time, these ADRs are diverse and nonspecific. It is important to note that combinations with other medications, such as antipsychotic, mood stabilizing, or anticholinergic drugs, have not led to an increase in ADRs in this population. Therefore, our findings clearly demonstrate the potential risks associated with prescribing non-benzodiazepine anxiolytic medications for more than 14 days in conjunction with sertraline for adolescents.

It is possible to draw conclusions that contradict the initially expected results. In girls experiencing a depressive episode, the presence of CYP2C19*17 polymorphism is associated with an increased frequency of ADRs when taking the antidepressant medication sertraline. However, the presence of CYP2C19*2 was not significantly linked to the safety profile of sertraline in these patients. The administration of non-benzodiazepine anxiolytic appears to be the most significant risk factor for the occurrence of ADRs in our sample.

Our study included few patients who took sertraline as monotherapy. However, this reflects the reality of treatment for depressive episodes in a typical urban hospital. Consequently, the results of our study not only replicate those previously shown by Rossow et al. (2020) paradoxical associations of CYP2C19*17 with sertraline safety (Rossow et al., 2020). Our study also confirms that it is important to study pharmacogenetic biomarkers together with other clinical factors. Often, the influence of other covariates (concomitant pharmacotherapy, child age, suicide attempts) may be more significant than the cytochrome P450 isoenzyme phenotype. This is all the more significant given that polypharmacy is a very common problem in pediatrics (Thiesen et al., 2013; Santos and Gondim, 2023). Future studies should include more patients and focus on issues related to the phenoconversion of cytochrome P450 isoenzymes. It is unlikely that the problem of polypharmacy will be overcome in the near future, but the need for personalized therapy selection is increasing. Pharmacogenetic studies should identify situations in which personalization based solely on patient genotyping will be ineffective.

### 4.1 Limitations

The study is observational and naturalistic, which reduces the credibility of the results obtained. Most patients were female, which limits the extrapolation of the results obtained. We performed genotyping of only *CYP2C19* polymorphisms, which limits the practical significance. The observation period was only 3 weeks, which allowed us to identify adverse reactions at the initial stage of pharmacotherapy. The small sample size is a likely reason for the insufficient statistical significance of the associations found. Most of the patients in the sample received additional pharmacotherapy; on the one hand, this does not allow us to assess the effect of sertraline monotherapy, on the other hand, we obtained data from real clinical practice, assessed the significance of various risk factors, and this increases the value of our results. We did not assess the severity of adverse reactions, which also limits the practical value of our results. In our study, steady-state plasma concentrations of sertraline were not measured, which does not allow us to assess the effect of the rate of CYP2C19 metabolism on the pharmacokinetics of the drug.

## 5 Conclusion

The carrier status of the CYP2C19*17 polymorphism is paradoxically more frequently associated with an increased risk of somatic and vegetative ADRs on the seventh day of treatment with sertraline in adolescent girls. The administration of non-benzodiazepine anxiolytics significantly increased the incidence of ADR complaints in adolescents on first and third weeks of taking pharmacotherapy. According to our findings, polypharmacy is a more significant risk factor for the development of ADRs in patients receiving sertraline, compared to the carrier status of *CYP2C19* polymorphisms.

## Data Availability

The original contributions presented in the study are included in the article/supplementary material, further inquiries can be directed to the corresponding author.
